# Seroprevalence studies on animal chlamydiosis amongst ruminants in five states of India

**DOI:** 10.14202/vetworld.2015.72-75

**Published:** 2015-01-24

**Authors:** R. Chahota, S. Gupta, B. Bhardwaj, P. Malik, S. Verma, and M. Sharma

**Affiliations:** Department of Veterinary Microbiology, DGCN College of Veterinary and Animal Sciences, Palampur, Himachal Pradesh, India

**Keywords:** agar gel precipitation test, chlamydiosis, ruminants, seroprevalence

## Abstract

**Background and Aim:**

Animal chlamydiosis, caused by different chlamydial species, is characterized by clinical or subclinical disease manifestations in cattle, buffalo, ovine, caprine and wild animal species. Animal chlamydiosis often remains underdiagnosed or undiagnosed, and its status in many parts of India is still unknown. Hence, the present study was conducted to determine the seroprevalence of animal chlamydiosis amongst ruminant livestock species of five states of India.

**Materials and Methods:**

Totally, 2127 randomly selected serum samples collected from ruminant livestock species *viz*. cattle (n=430), buffaloes (n=429), sheep (906) and goats (n=362), were tested by agar gel precipitation test for chlamydiosis between 2002 and 2011. Precipitating antigen was prepared from locally isolated strain of *Chlamydia psittaci* after treatment with sodium deoxycholate.

**Results:**

The chlamydial seroprevalence detected amongst ruminants in five states of India was: Himachal Pradesh: Cattle-10.90%, sheep-10.60% and goats- 22.46%; Punjab: Cattle-1.45%; Andhra Pradesh: Cattle-2.80%, buffaloes-0.93%, sheep-8.90% and goats-9.46%; Maharashtra: goats-8.33%; Jammu and Kashmir: sheep-12.50%. The mean seroprevalence values of each animal species are: Cattle-4.65%, buffaloes-0.93%, sheep-9.82% and goats-19.33%.

**Conclusion:**

The results indicate the endemic nature of animal chlamydiosis across five states in India. Hence, it requires further extensive studies in other parts of India also using chlamydial species-specific diagnostics to ascertain overall countrywide prevalence of the disease. The zoonotic nature of the chlamydiae of ruminant origin further adds significance to such prevalence studies.

## Introduction

Chlamydiae are obligate intracellular bacterial pathogens, responsible for diverse disease conditions in animals, birds and humans [1-3]. Chlamydiae undergo a unique biphasic developmental cycle characterized by two morphologically distinct forms called the elementary body (EB) and the reticulate body. Chlamydiae, a genetically diverse group of organisms belonging to order *Chlamydiales*, family *Chlamydiaceae* were grouped into two genera, *Chlamydia* and *Chlamydophila*, containing nine species [4]. But recently, a single genus classification (*Chlamydia*) with 11 species has been adopted by International Committee on Systematics of Prokaryotes [5] and is currently being followed [2-6]. Animal chlamydial infections are caused by different chlamydial species of the genus *Chlamydia*. Amongst ruminants, *Chlamydia abortus, Chlamydia pecorum* and *Chlamydia psittaci* cause clinical infections [7-9]. *C. abortus* causes abortion, infertility and weak offspring in sheep, goats, cows and buffaloes, whereas, *C. pecorum* is associated with chronic conditions such as pneumonia, polyarthritis, conjunctivitis, enteritis, encephalomyelitis, metritis and mastitis amongst ruminants. *C. psittaci* mainly causes “avian chlamydiosis” amongst 471 reported avian species [10] but can also infect mammalian species including ruminants [9]. Chlamydiae are also accountable for direct zoonosis (animals to humans) without the involvement of any intermediate host and some species like *C. psittaci*, *C. abortus*, *Chlamydia caviae* and *Chlamydia felis* are confirmed zoonotic pathogens [11,12].

Chlamydial diseases amongst ruminants are known in India since long time [13,14] but as the result of using improved diagnostic techniques in recent years, chlamydiosis has been recognized as one of the important health problems in livestock [15-17]. Recent serological surveys carried out in many countries to know the status of chlamydiosis in native livestock species using different screening tests have revealed the prevalence of this disease to variable extent in both diseased and normal animals [17-19]. In India, the prevalence of animal chlamydiosis is well studied in only few areas but little is known about its status in other states due to the difficulty associated with the handling of this fastidious intracellular pathogen.

Hence, the present study was undertaken to investigate the seroprevalence of animal chlamydiosis in five states of India.

## Materials and Methods

### Ethical approval

Prior consent of the owners of the animals was taken before collection of blood samples for serum extraction. Proper ethical considerations related to animal handling were observed and ensuring not to cause any injury during sampling. The other institutes that submitted the serum samples for diagnosis of the chlamydiosis addressed the animal ethics issues according the local regulations.

### Samples

The study was conducted during the period of 2002-2011 using 2127 serum samples collected randomly or submitted to the Department of Veterinary Microbiology for chlamydial diagnosis from populations of ruminant livestock species from five different states such as Himachal Pradesh, Punjab, Jammu and Kashmir, Andhra Pradesh and Maharashtra. The detail of sample collection is shown in Table-1.

**Table 1:** Details of the samples collected from small and large ruminants

Name of the state	Cattle	Buffaloes	Sheep	Goats	Total
Himachal Pradesh	110	-	415	276	801
Punjab	69	-	-	-	69
Andhra Pradesh	251	429	467	74	1221
Maharashtra	-	-	-	12	12
Jammu and Kashmir	-	-	24	-	24
Total	430	429	906	362	2127

### Chlamydial group specific antigen preparation

Chlamydial group specific antigen was prepared from locally isolated *C. psittaci* strain from the placenta of an aborted sheep that was multiplied in McCoy cell line. The cells were maintained in Eagle’s minimum essential medium (Gibco) containing 5% fetal bovine serum and 10 mg/ml of gentamicin and 1 mg/ml of cycloheximide and incubated at 37°C in 5% CO_2_. The EBs were harvested 3-4 days after inoculation and were partially purified. AGPT antigen was prepared by treating these EBs with sodium deoxycholate as reported earlier [20].

### Chlamydia specific positive serum

Positive serum used in test proper was raised in rabbit using above-mentioned strain of *C. psittaci* as described earlier [21]. Briefly, 100 mg of EB protein was treated with 1% Triton X-100 in 1 ml of phosphate-buffered saline at 37°C for 1 h and sonicated. The EBs were emulsified with an equal volume of Freund’s complete adjuvant (Sigma) and given intramuscularly. After 3 weeks, a series of five intravenous injections of treated antigen were given at 3 days intervals for the first four injections and 7 days for the fifth, in progressively increasing doses (10, 10, 15, 20, and 50 mg of protein). Rabbits were bled 1 week after the last injection. Hyperimmune serum was suitably diluted for using in test proper.

### Agar gel precipitation test (AGPT)

The AGPT test was performed as described earlier [22] with minor modifications using 1% agar in normal saline solution. Briefly 4-5 ml of molten agar was pipetted onto microscope slides. Wells of 4.0 mm diameter were cut into the hardened agar at 3-4 mm distance. The slides were incubated at 4°C in a humidified chamber and appearance of whitish lines of the precipitation reaction in the test and control samples were recorded after 16-24 h. The antigen-antibody reaction in the form of clear precipitation line was observed in chlamydia positive cases. Negative and positive serum controls were also kept along with each test.

### Statistical analysis

The hypothesis that the seroprevalence of chlamydiosis in animal populations of different states is same was tested using c^2^ test function in Microsoft Excel software at 5% (0.05<p) significance level and results of observed prevalence (experimental hypothesis) were analyzed.

## Results and Discussion

Chlamydial infections have been reported from different livestock species globally [2,23,24]. In India, chlamydiae are reported to cause abortions, infertility, conjunctivitis, pneumonitis, enteritis and encephalomyelitis amongst cattle, sheep, goats and equines [12-16]. Though direct evidence of the infectious agent is the ultimate diagnosis, sero-assays are more suitable for screening large numbers of population samples especially when samples are to be analyzed in another laboratory. Serodetection of circulating antibody is a convenient method for detecting infectious diseases. Seronegative results would indicate no previous contact with chlamydiae, whereas, seropositive test indicates an active infection in test animal or being a potential carrier of the disease. Different serological tests for the detection of chlamydial infections are reported such as microimmunoflorescence, enzyme-linked immunosorbent assay, AGPT, complement fixation test and indirect hemagglutination assay. These tests differ in their sensitivities and specificities, depending primarily on the antigen preparation, and all the applied serological methods can be widely used in laboratories as diagnostic methods for establishing infections amongst ruminants with *Chlamydia* spp. Though, AGPT is less sensitive but does not require any specialized infrastructure and can easily be performed under in house conditions. AGPT is reported to detect chlamydial complement fixation antibody titer of more than 8, which is considered advantageous in ruling out weak reactors in case of field studies [22].

Analysis of disease-related data from five states revealed that seroprevalence was highest, i.e. 19.33% amongst goats. However, seroprevalence of chlamydiosis amongst goats of different states of India showed variations from 8.33% in Maharashtra to 22.46% in Himachal Pradesh, and the detail is shown in Table-2. The low positive percentage in Maharashtra may be due to small sample size. Amongst sheep population, we detected a mean prevalence of 9.82%, ranging from 8.9% in Andhra Pradesh to 10.6% in Himachal Pradesh. Studies on limited samples and area in India by Mahapatra *et al*. [25] using AGPT for chlamydiosis, recorded 28.0% and 34.0% seropositivity for sheep and goat, respectively. Similarly, Joshi *et al*. [26] also reported seropositivity of 29.90% and 43.29% in sheep and goats, respectively. In cattle and buffaloes, overall 4.65% and 0.93% samples were found seropositive, respectively in our study. We tested 429 samples of buffaloes from Andhra Pradesh only; however, it requires further studies of buffalo samples from other parts of India also. In one of our previous studies on buffalo serum using AGPT, total 8.70% samples were found positive from Himachal Pradesh [14]. In this study, no significant statistical difference was found in the seroprevalence of chlamydiosis amongst ruminants of five different states of India. This may be due to the small size of the representative samples from each state. Thus, a more exhaustive epidemiological study is required to know the actual chlamydial prevalence in different states. Though, the present investigation underlines the importance of the animal chlamydiosis on the health of livestock in India. It is observed that seropositivity for chlamydiosis varies frequently amongst animals because of several influencing factors including the degree of exposure to infection, feeding habitats, virulence of chlamydial strains and possibly innate immunity amongst animal species. But for population-based studies to determine the prevalence of any disease this approach is indispensable. Therefore, recently numerous serologic surveys on the prevalence of chlamydiosis amongst ruminants have been carried out in other countries to ascertain the effect of chlamydiosis on animal health and production [17,18,27-29]. Thus, in India, extensive epidemiological studies are required to be undertaken including more livestock populations of different geographical locations of the country for better understanding of animal chlamydiosis.

**Table 2:** Seroprevalence of chlamydial infections amongst ruminants

Name of the state	Cattle (%)	Buffaloes (%)	Sheep (%)	Goats (%)	Total (%)
Himachal Pradesh	12/110 (10.90)	-	44/415 (10.60)	62/276 (22.46)	118/801 (14.73)
Punjab	1/69 (1.45)	-	-	-	1/69 (1.45)
Andhra Pradesh	7/251 (2.80)	4/429 (0.93)	42/467 (8.90)	7/74 (9.46)	60/1221 (4.91)
Maharashtra	-	-	-	1/12 (8.33)	1/12 (8.33)
Jammu and Kashmir	-	-	3/24 (12.5)	-	3/24 (12.5)
Total	20/430 (4.65)	4/429 (0.93)	89/906 (9.82)	70/362 (19.33)	183/2127 (8.60)

Note: Positive percentages are shown in the parenthesis

## Conclusion

The results of the present investigation indicated that chlamydial infections are endemic amongst ruminants in studied areas of five states of India, which may represents one of the causes of abortion, pneumonia, enteritis, arthritis and other clinical manifestations. Therefore, large-scale screening of native livestock species along with characterization of involved indigenous chlamydial strains is urgently required. Thus, integrated strategies are required to be adopted for control and prevention of sporadic chlamydial infection cases or disease outbreaks keeping in view the zoonotic importance also.

## Authors’ Contributions

RC was associated with overall planning and execution of the research work. SG, BB and PM carried out laboratory work. SV and MS analyzed the data and drafted and revised the manuscript. All authors read and approved the final manuscript.
